# Caesarean section deliveries: Experiences of mothers of midwifery care at a public hospital in Nelson Mandela Bay

**DOI:** 10.4102/curationis.v41i1.1804

**Published:** 2018-01-30

**Authors:** Thobeka P. Jikijela, Sindiwe James, Balandeli S.I. Sonti

**Affiliations:** 1 Department of Nursing Science, Nelson Mandela Metropolitan University, South Africa

## Abstract

**Background:**

The rate of caesarean section deliveries has increased globally and mothers are faced with challenges of postoperative recovery and caring thereof. Midwives have a duty to assist these mothers to self-care.

**Objective:**

The objective was to explore and describe experiences of post-caesarean section delivered mothers of midwifery care at a public hospital in Nelson Mandela Bay.

**Methods:**

A qualitative, descriptive and explorative research design was used in the study. Data were collected from 11 purposively criterion-selected mothers who had a caesarean section delivery. One-on-one semi-structured interviews were conducted in the post-natal wards. Research ethics, namely autonomy, beneficence, justice and informed consent, were adopted in the study. All participants were informed of their right to withdraw from the study at any stage without penalties. Interviews were analysed using Tesch’s method of data analysis.

**Results:**

Three main themes were identified as experiences of: diverse pain, physical limitation and frustration and health care services as different.

**Conclusion:**

Experiences of mothers following a caesarean section delivery with midwifery services at a public hospital in Nelson Mandela Bay were explored and described as diverse. A need for adequate pain management as well as assistance and breastfeeding support to mothers following caesarean delivery was identified as crucial to promote a good mother-to-child relationship.

## Introduction

Having a baby is usually a happy and exciting moment for delivering women. They are excited to hold their babies, feed them and provide care for them (Ismail, Shahzad & Shafiq 2012:36). However, for some women, this excitement is dampened as there is physical pain, especially for those women who have had a caesarean section delivery. They experience discomfort as they struggle to cope with the pain from the caesarean section wound. Their pain also limits the interaction between them and the baby and also causes the mothers to be reluctant to breastfeed (Vercauteren 2010:130). In some cases, the baby is admitted to the nursery and visiting the baby is difficult for the mother because of the pain. These mothers by rule need to bond with their neonates as early as possible and begin breastfeeding (Bamigboye & Hofmeyer 2012:314). Furthermore, taking care of their infants at home after discharge from the hospital can also be a challenge. However, pain that is well managed facilitates mother-to-child bonding, more effective infant care and improved breastfeeding, and also limits the stay of the infant and the mother in the hospital (Karlstrom et al. 2010:1330).

## Problem statement

A caesarean section delivery has become a regular practice around the world when an obstetrical complication is envisaged. The concern is, however, the pain and immobility associated with the surgery postoperatively as well as the impact it might have on the caring of the neonate. At a referral hospital in the Nelson Mandela Bay, Eastern Cape, mothers breastfeed as soon as they deliver, take care of the neonates and are also expected to visit and feed the neonates if they are admitted to a neonatal unit. However, mothers who had a caesarean section delivery can only start mobilising freely after 24 h. This immobility is associated with insufficient pain control or excessive sedation (Sharma et al. 2012:462). However, as the researcher also at times does some contract work at the local private hospitals, it is observed that mothers were on continuous intravenous low-dose sedation for at least 12 h postoperative.

It is assumed to be difficult for them to adopt a comfortable sitting position to breastfeed or even to express breast milk to feed the neonate because of postoperative pain. When mothers cannot breastfeed, their babies are predisposed to hypoglycaemia if there are no other means of feeding, therefore needing further treatment. Furthermore, hospital stay of both the neonate and the mother will be extended leading to financial implications for the institution.

## Aim of the study

The aim of the study was to explore experiences of post-caesarean section delivered mothers of midwifery care at a public hospital in Nelson Mandela Bay. Furthermore, based on the results of the study, make recommendations that will assist midwifery operational managers and midwives to assist mothers following a caesarean section delivery to manage their pain better.

## Background

There has been an increase in the rate of caesarean section deliveries of approximately 18.5 million each year despite the World Health Organization (WHO) guidelines stating that the caesarean section rate should not exceed 15% of the total deliveries per year per country (Rauch 2011:428). Currently, in South Africa, the caesarean section rate has increased to 22.5% between 2012 and 2013 (Pattinson & Rhoda 2014) and about 70% of these deliveries are conducted from the private sector (Monticelli 2013:4). The major concern mainly being that some of the caesarean sections may be done without medical or obstetric indication (Boutsikou & Malamitsi-Puchner 2011:1519).

A caesarean section delivery involves major surgery and it is therefore necessary to ensure extensive pain management to help make the woman reasonably comfortable during the post-delivery period (Karlstrom et al. 2010:1330). Proper management of pain following a caesarean section delivery can reduce a hospital stay by 50% (Vercauteren 2010:129). Mothers who have had a caesarean section delivery only start mobilising freely after 24 h compared to those who have had a vaginal birth (Boutsikou & Malamatsi-Puchner 2011:1520). This immobility is associated with insufficient pain control (Sharma et al. 2012:462).

A caesarean section delivery is major surgery that involves a lot of muscle stretching and it is therefore necessary to ensure extensive pain management to make the woman reasonably comfortable during the post-delivery period (Karlstrom et al. 2010). Analgesics that are commonly used are pethidine, coupled with non-steroidal anti-inflammatory drugs (NSAIDs) (Ismail et al. 2012:40). NSAIDs have been shown to reduce pain caused by uterine muscle cramping (Vercauteren 2010). Proper management of pain following a caesarean section delivery can reduce a hospital stay by 50% (Vercauteren 2010). The regime should be safe, highly effective and at the same time allow for a quick recovery, immediate interaction with the baby and a reduced chance of being transmitted to the neonate via breast milk (Rauch 2011); therefore, pain medication should be carefully chosen to promote early recovery and allow mothers to breastfeed, take care of their neonates and be discharged soon.

The principal objective of the study conducted was to establish an understanding and further describe how mothers experienced caring for their neonates following a caesarean section delivery. After data collection and analysis literature had been reviewed to compare findings, the findings revealed that after a caesarean section delivery, women experienced pain differently for several reasons such as the quality of their support systems, emotions and pain. A few studies were found that reported similar findings (Redshaw 2010:150; Sharma et al. 2012:462).

In the current study, it was found that mothers also experienced pain as an achievement of motherhood. It was also revealed in the findings that mothers could not take care of themselves and their babies because of physical limitations that made it difficult to breastfeed. These findings were also confirmed in a study conducted by Van Reenen and Van Rensburg (2013:271) and Weiss, Fawcett and Aber (2009:2940). Other findings were that participants were dissatisfied with maternity services and the health workers’ attitudes. Several other studies also came up with similar findings (Lumadi & Buch 2011:26; Keedle et al. 2015:10). These studies were focusing on the satisfaction with services and included vaginal deliveries, whereas this study focused on experiences of caring for the baby immediately following a caesarean section. No other studies have been found regarding this issue.

Although caesarean section deliveries are common throughout the world, these operations carry certain risks and can affect both the mother and the neonate negatively. Hence, it is critical that the pain experienced by the mother after the operation is managed effectively, because it can affect both her health and that of her neonate.

### Trends

Several studies have been conducted previously on the topic which include findings that participants were dissatisfied with maternity services and the health workers’ attitudes. Similar findings were also found in the current study. Several other studies (Lumadi & Buch 2011:26; Keedle et al. 2015:10) were focusing on the satisfaction with services and included vaginal deliveries, whereas this study focused on experiences of caring for the baby immediately following a caesarean section. No other studies have been found regarding this issue.

## Research objectives

To explore and describe the experiences of post-caesarean section delivered mothers of midwifery care at a public hospital in Nelson Mandela Bay in order to formulate recommendations to assist midwifery operational managers and midwives to support mothers who have undergone a caesarean section delivery to manage their pain.

## Definition of key concepts

**Caesarean-section delivery**: is a major surgical procedure by which a baby is delivered through a surgical incision in the abdominal wall and uterus. It may be done under general epidural or spinal anaesthesia (Dippenaar & Da Serra 2013:482). In this study, a ‘caesarean-section delivery’ refers to the surgical operation by which babies were delivered to the mothers who will participate in this study.

**Experiences**: refer to the events or facts that a person has encountered and observed that leave a lasting impression (Soanes & Stevenson 2008). In the current study, experience refers to the feelings, perceptions and attitudes of mothers with regard to caring for their neonates following a caesarean section delivery.

**Mothers**: are female parents (Soanes & Stevenson 2006:589). For the purpose of this study, the term ‘mother’ refers to a woman who has given birth to a live baby via a caesarean section delivery.

**Midwife**: is a person who, having been regularly admitted to a midwifery educational programme, duly recognised in the country in which it is located, has successfully completed the prescribed course of studies in midwifery and has acquired the requisite qualification to be registered or legally licensed to practise midwifery (Sellers 2012:12). In the context of this study, a midwife shall refer to the registered nurse working in the postnatal ward, nursery or clinic and providing care and support to delivered women and neonates in the nursery.

## Contributions to the field

The study results will assist caesarean section delivered mothers to cope better with pain and taking care of their neonates following the delivery. Also, the results will provide recommendations to assist midwifery operational managers and midwives to support mothers who have had a caesarean section delivery in managing their pain.

## Research method and design

### Research design

The study used a qualitative, explorative, descriptive and contextual design. A qualitative research design provides an in-depth examination of qualities and characteristics of a phenomenon so as to understand and explain it better (Botma et al. 2010:182). Furthermore, it is stated that qualitative research aims to study people in their natural social settings and collect naturally occurring data (Creswell 2013:191). The current study aimed to understand and describe the experiences of mothers of midwifery services at a public hospital in Nelson Mandela Bay.

### Population and sampling

The research population were mothers who had a caesarean section delivery in a public health institution in the Nelson Mandela Metropolitan Municipal area in the Eastern Cape Province. Purposive and sequential sampling was used to select participants in the study. A sample of 11 mothers was purposively selected (Botma et al. 2010; Kumar 2014) using set criteria (Botma et al. 2010; Burns & Grove 2011; De Vos et al. 2011).

### Data collection method

Data were collected using one-on-one, semi-structured audio-captured interviews, field notes and observations. The recording was necessary to capture all the information to facilitate transcription of the interview data verbatim. The average duration of each interview was 40 min and data collection continued until data saturation. The question asked was:

Can you tell me about your experience of midwifery care following your caesarean section delivery operation?

A predetermined interview schedule was used and the questions asked were:

How was your experience of caring for your baby following your caesarean section delivery operation?

How did you feel about the pain medication and care that you received following your caesarean section delivery?

What influence did your caesarean section delivery have on breastfeeding and caring for your neonate?

Eleven interviews were conducted. Of these 11 interviews, two were from the pilot study. Because there were minor errors to be attended to in the pilot study and the methodology remained the same, it was suggested to include the pilot study in the main study and analysis.

### Data analysis

Data analysis was continuous with data collection (Creswell 2013:191) but formally started as soon as the interview phase was completed using Tesch’s method of analysis-transcribed interviews. An independent coder was used to assist with data analysis.

All the recordings were listened to immediately after the interview and rewound as far as possible to understand the participant’s perspectives of their experiences in their own words.

Then, a verbatim transcription of each recording was done as soon as possible to capture all the important information.

Thereafter, all the field notes and transcriptions were read through to get sense of all the information. Then, all the information was organised to facilitate analysis using a process of coding (Creswell 2013:189). An independent coder was used to assist with finalising coding of themes and sub-themes and to interpret the meaning of the findings and counter any bias from the researcher (Creswell 2013:191). An appointment for a consensus meeting between the researcher and the independent coder was fixed and the results finalised. A report was written on the results and submitted to the research supervisor for comments. Interview transcripts and field notes hard copies were locked away for safekeeping in case they are needed later.

### Context of the study

The study was conducted in the period of April 2015–August 2015 in postnatal and neonatal units at a public hospital in the Nelson Mandela Bay, Eastern Cape. The institution was chosen because it is a referral hospital for the Nelson Mandela Bay and the surrounding district hospital where most caesarean section deliveries are conducted. A private side ward was used for conducting interviews to ensure privacy.

### Potential benefits and hazards

The study was approved by the Faculty of Health Sciences’ postgraduate student committee at the Nelson Mandela Metropolitan University. Ethical clearance was also given by the same committee (H14-HEA-NUR-207).

Participants were informed that there will be no incentive for participating in the study but future benefit for mothers in improving nursing care and support given to make them more comfortable following a caesarean section delivery. Participants were also informed that no names will be used and instead will be replaced by codes to protect them from harm.

### Recruitment procedures

Participants were identified and selected with the assistance of the nurse managers and midwives working in identified units who supplied the researcher with the necessary admission and delivery register. Participants were approached individually by the researcher, making sure of privacy. Participants were informed that they could take part only if they are willing to and that they could withdraw during the study when they feel uncomfortable without any negative consequences.

### Informed consent

Each participant was briefed about objectives and the purpose of the study and the reason for selecting them. Clear and accurate information was given to the participants using simple language. After agreeing to participate in the study, consent form was signed and the mothers were reminded that they can withdraw at any stage of the study if they feel uncomfortable.

### Data protection

Results from the pilot study, audio-recorded information, transcribed audio-recorded information and field notes were kept under lock and key so that no other people can access the information. Also, no names were used when transcribing the information and addresses and institution were replaced with pseudonyms.

### Trustworthiness

Trustworthiness is a mean of checking the accuracy and consistency of the findings by using certain procedures (Botma et al. 2010:231). Trustworthiness was maintained throughout the study using the four standards namely: truth value, applicability, consistency and neutrality.

### Truth value

Truth value means that the study is conducted in a truthful manner and the findings are presented as experienced and lived by the participants (Botma et al. 2010:233). Truth value was obtained by using the strategy of credibility.

### Credibility

The aim of credibility is to demonstrate that the study is conducted in such a way as to ensure the participants have been identified and described accurately (De Vos et al. 2011:420). To increase the credibility of the study, the researcher used the following activities.

### Triangulation

Triangulation is the use of different data sources of information by examining evidence from the sources and using it to build themes (Botma et al. 2010:231). In this study, the researcher did an extensive literature review using different books and journal articles to obtain different points of view.

### Member checking

Member checking means that the researcher takes the results back to the participants to clarify mistakes and confirm that the findings reflect what they said (Schmidt & Brown 2012:356). For the purpose of this study, the participants were given an opportunity to clarify and confirm their responses while they were being interviewed.

### Peer debriefing

Peer debriefing occurs when regular meetings are held with colleagues and experts who are not involved in the study in order to discuss the methods and findings of the study (Flick 2009:392). The researcher arranged a meeting with the research supervisor to discuss the findings of the research.

### Applicability

Applicability refers to the degree to which the findings can be applied to different contexts and groups (Botma et al. 2010:233). Applicability uses transferability as a strategy.

### Transferability

Transferability means that the findings of the study can be transferred to a similar context to establish trustworthiness (Schmidt & Brown 2012:354). It uses the following activities.

### Thick or dense description

Thick or dense description means that the researcher will do a complete detailed explanation of the research context, participants, methodology and interpretation to enhance the reader’s understanding (Merriam 2009:43). In this study, the researcher described in detail the methodology, the setting, how data were collected and analysed to enable other researchers to transfer the findings to a similar study.

### Data saturation

Data saturation is achieved when the participants provide no new information and the themes become repetitive (Schmidt & Brown 2012:354). In this study, data saturation was confirmed when the participants came up with no new information even with probing and no new themes were developed on analysis.

### Purposive sampling

Purposive sampling maximises specific information obtained from and about the particular context by purposefully selecting participants who have knowledge of the phenomenon under study (Brink, Van der Walt & Van Rensburg 2012:173). The researcher selected only mothers who had a caesarean section delivery as it was the researcher’s perception that they had the knowledge and experiences with regard to pain management when caring for their neonates. A set inclusion criteria enhanced the purposive sampling.

### Consistency

Consistency considers whether the study will yield the same results if the inquiry was replicated with the same participants in a similar context. Consistency uses dependability as a strategy (Botma et al. 2010:233).

Dependability is the extent to which the study produces the same results when repeated in a similar context with the same participants (De Vos et al. 2011:420). The researcher used the following activities.

### Dependability

Dependability involves an audit to describe in detail data collection methods, process and analysis and submit to an external auditor to check if the process is logical and well documented (De Vos et al. 2011:420). In this study, the researcher used an auditor to check the processes of data collection and analysis.

### Peer examination

Peer examination involves discussing the results, recommendations and the process of the study with the co-facilitator, the supervisor and colleagues who are not involved in the study. The findings and recommendations were discussed with the research supervisor.

### Co-coder and independent coder

The researcher made use of a co-coder (the supervisor) and an independent coder to evaluate the process of the research and establish whether they agreed with the concepts and themes.

### Thick description

Thick description involves the researcher giving a full and detailed description of how the data were collected and analysed and thereafter submitted to an independent coder. On completion of data collection and analysis, a full and detailed description was submitted to the research supervisor and an independent coder.

### Neutrality

Neutrality is the degree and extent to which findings are based solely on an interpretation of the participants and conditions of the research, and not on other biases, motives or perspectives (Botma et al. 2010:233). Neutrality uses the strategy of confirmability.

### Confirmability

The researcher needs to ask whether the findings of the study could be confirmed by another study and provide evidence that links the findings and interpretation (De Vos et al. 2011:421). In this study, the researcher used the following activities.

### Confirmability audits

Confirmability audits involve the forwarding of themes and sub-themes to the independent coder for confirmation. In this study, different sources of information and literature were used to confirm the data that were collected. Also, field notes and audio recordings were checked and verified with the supervisor.

### Reflexivity

The researcher made use of reflexivity, which is the process of reflecting on self and identifying personal values that could affect data analysis and interpretation of the findings (Polit & Beck 2012:179). In the current study, reflexivity was achieved by holding discussions with the independent coder to prevent biases, thus increasing objectivity.

Participants were given an opportunity to confirm that the data had been transcribed accurately.

### Presentation of results

Results of the study are presented in Tables 1 and 2.

**TABLE 1:** Demographic profile of participants.

Item number	Age in years	Nationality	Gravidity	Parity	Language	Marital status
1	22	Mixed race	2	2	Afrikaans	Single
2	29	African	2	1	English	Single
3	39	Black	3	5	Xhosa	Single
4	20	Black	1	1	Xhosa	Single
5	27	White	1	1	English	Single
6	27	Mixed race	2	2	Afrikaans	Married
7	25	Black	1	2	Xhosa	Single
8	29	Black	2	2	Xhosa	Single
9	26	Black	1	1	Xhosa	Single
10	18	Mixed race	2	1	English	Single
11	21	Mixed race	2	2–1	Afrikaans	Single

*Source*: Authors’ own work

**TABLE 2:** Themes and subthemes of the study.

Themes	Sub-themes
1. Mothers had diverse experiences of post-caesarean section pain.	Pain had been experienced as fulfilling, unbearable, manageable through pain-relief medication
2. Mothers had experienced pain after caesarean section delivery as frustrating and physically limiting.	The pain caused: numbness, difficulty in breastfeeding, limitations to self-care
3. Health care services had been experienced differently.	Services experienced as: neglectful and uncaring, supportive, disappointing

*Source*: Authors’ own work

## Discussion of results

Mothers experienced the care post-delivery differently.

### Theme 1: Mothers had had diverse experiences of post-caesarean section pain

The theme described various experiences of pain by mothers following a caesarean section delivery.

#### Sub-theme 1.1: Pain had been experienced as fulfilling

As reported by Van Reenen & Van Rensburg (2013:272), motherhood following an unplanned caesarean section delivery had included feelings of deep affection and emotional bonding with their neonates. Participants described having felt a loving and caring concern for their babies and had regarded pain as a worthy gift. Such responses are as follows:

‘… But it (the pain) was nothing and to be a mom is the best thing in life that can ever happen to you.’ (Participant 5, Grav1 Para1, 27 years old)

‘It was the best experience for me because I’ve always wanted to be a mom really.’ (Participant 9, Grav1 Para1, 26 years old)

Participants in this study embraced motherhood and the gift of having a live healthy baby, and therefore pain was seen as the most rewarding feeling and could be tolerated. These participants were emotionally satisfied from having a live baby and had responded positively by focusing more on their neonates which made them forget about the pain.

#### Sub-theme 1.2: Post-caesarean-section pain had been experienced as unbearable

Pain following caesarean section delivery is said to be one of the most frequently reported problems in obstetrics (Chin, Vincent & Wilkie 2014:279; Karlstrom et al. 2010:1336). Some of the participants reported that they had experienced post-operative pain as extremely strong and stabbing.

‘Oh it was painful because it felt like I was being pulled from the operation site.’ (Participant 1, Grav2 Para2, 22 years old)

‘Oh it felt like I was being stabbed so many times. It felt like they left the knife there.’ (Participant 8, Grav2 Para2, 28 years old)

Furthermore, for them being given their babies as soon as they came back from theatre was unexpected.

‘Wait let me tell the truth that moment I was in pain I was wishing for the baby to be taken away so I can be able to handle the pain first.’ (Participant 9, Grav1 Para1, 26 years old)

#### Sub-theme 1.3: Post-caesarean section pain had been experienced as being manageable through pain-relief medication

Results from the study of Karlstrom et al. (2010:1331) revealed that women whose post-operative pain was well managed are able to breastfeed and mobilise and their hospital stay is shortened, while the postnatal period is comfortable and emotionally satisfying for the mothers (Bamigboye & Hofmeyr 2012:1). Responses to that effect were:

‘It (*the pain medication*) is helpful and they also gave the injection. I think I took the injection twice only then I was able to handle the pain.’ (Participant 1, Grav2 Para2, 22 years old)

### Theme 2: Mothers experienced post-caesarean section pain as frustrating and physically limiting

For some of the participants, the pain had affected the transition to motherhood and the ability to perform certain activities and had therefore hindered them from caring for themselves and their neonates.

#### Sub-theme 2.1: Mothers experience numbness

Spinal anaesthesia can result in temporal loss of sensation but could also result in a loss of control in the upper limbs, therefore causing difficulty in holding the baby (Zwedberg, Blomquist & Stgerstad 2014:218) and make mothers feel disconnected from their bodies (Kuguoglu et al. 2012:125; Van Reenen & Van Rensburg 2013:272).

‘You can’t feel yourself, it feels like there is nothing there (*pointing the feet*) like you, you want to bend it but you don’t know if it’s bending or not, your toes.’ (Participant 2, Grav2 Para1, 29 years old)

#### Sub-theme 2.2: Pain caused difficulty in breastfeeding

In South Africa, breastfeeding is the most important part of being a mother and assists with meeting health priorities stipulated by the South African Government (Pound & Unger 2012:317). Participants mentioned having experienced difficulty in breastfeeding following a caesarean section delivery especially on their first day.

‘My breasts are sore but I can’t take it out of the mouth because of that pain.’ (Participant 3, Grav3 Para5, 39 years old)

Participants had undergone caesarean section under spinal anaesthesia and therefore had to lie flat for at least 6 h, but their neonates were brought to them while they were still lying flat. The experience was especially difficult for the participants who had twin babies as evidenced in the statements below:

‘I tried [*to breastfeed*] but I could not manage well. … I could not pick them [*twins*] up.’ (Participant 3, Grav3 Para5, 39 years old)

Although some mothers had enjoyed bonding with their babies, some reported that they had needed to get better before engaging with their neonates.

#### Sub-theme 2.3: Mothers had experienced limitations to self-care

For some of the participants, the painful recovery period from caesarean section delivery had been extremely demanding mostly because of the physical limitations accompanying it. According to McGrath and Phillips (2009:10), limited self-care makes the mother feel incompetent and doubts her ability to care and provide for her neonate (Crowe et al. 2008:237; Declerq et al. 2008:17). Participants in the study expressed the fact that they could not walk, cough, sit up and care for the neonates.

‘It’s like you can’t stand on your feet or walk. It’s like your legs and everything doesn’t want to move.’ (Participant 8, Grav2 Para2, 29 years old)

The mothers had not only been faced with limitations to self-care but also with the new responsibilities of motherhood; therefore, it had been difficult for them to perform all these tasks simultaneously.

### Theme 3: Health care services had been experienced differently

In order to reduce the global burden of maternal mortality and morbidity health care providers, facilities and the health-care systems should promote respectful midwifery care (Karim, Goni & Murad 2013:425; Kruger & Schoombee 2010:84; Moyer et al. 2014:263).

#### Sub-theme 3.1: Neglectful attitude of midwives experienced as uncaring

Respectful care is a key component of a mother-friendly hospital birth initiative and a vision of the WHO (WHO & UNICEF 2009:2). Participants expressed a concern that the care and assistance they had received was not what they expected, but uncaring, neglectful and disappointing.

‘I just came back from theatre and I was given 2 babies and when I asked for help because I wanted to breastfeed, I was told to sit up.’ (Participant 7, Grav1 Para 2, 25 years old)

‘They just put the baby, just call you to say this is your baby you take the baby and breastfeed and leave you just like that. Hey, they do have a tendency of not caring a lot, they do not care much.’ (Participant 6, Grav2 Para 2, 27 years old)

‘You cannot whilst you in pain just been cut, in the mean time someone is telling you to sit up, not even closer to see if you can really do that.’ (Participant 11, Grav2 Para 2-1, 21 years old)

Mothers rely on midwives for support and advice. They come with expectations and if these are not met, their disillusionment may affect their use of health care services in future.

#### Sub theme 3.2: Services had been experienced as being supportive

The law holds that the right to health care needs services that are available and supportive to women’s reproductive health (Bohren et al. 2014:12). Satisfaction with health care services also makes them feel pleasant and promotes mental health (Changee et al. 2015:21). Some of the participants expressed receiving positive support from midwives especially with regard to their breastfeeding problems.

‘My breasts were sore, I showed the sister so she showed me how to breastfeed, like how to put her properly on the breast and it’s not sore now.’ (Participant 5, Grav1 Para1, 27 years old)

‘I was shown and taught how to breastfeed on the first day.’ (Participant 4, Grav1 Para 1, 20 years old)

Successful breastfeeding improves mother and child bonding and is experienced as an achievement of motherhood (Zwedberg et al. 2014:216).

Because of the support they received, these participants had felt empowered and had developed a better relationship with their neonates. This is congruent with Lumadi and Buch’s (2011:24) study results.

#### Sub-theme 3.3: Health services had been experienced as disappointing

Health care organisations and health care workers have a duty to care and provide services that are accessible, acceptable and of good quality to health users (Bohren et al. 2014:17; Freedman et al. 2014:915). The expectations and experiences are consistent with the results in the current study.

‘I was comfortable with my first twins … but now things have changed in as much that I feel sorry for those who must still come and deliver after me.’ (Participant 7, Grav1 Para2, 25 years old)

‘Seems like we must follow a certain rule and we just have to follow and if ever those people from the Government can experience this, they would not like it themselves.’ (Participant 1, Grav2 Para2, 22 years old)

The hospital equipment had been perceived as a hindrance in the mother’s ability to care for their babies. Low quality or defective hospital equipment is not only uncomfortable but also makes it difficult for the workers to perform their duties properly and can lead to injuries (Bohren et al. 2014:13; Mosadegharad 2014:78). Participants expressed that their concerns had been more about their safety and that of the babies.

‘But this bed is really uncomfortable.’ (Participant 6, Grav2 Para2, 27 years old)

‘When you wake the baby can fall off the bed and some of us we are not even used to single beds and if you lying on one side you cannot move at all.’ (Participant 4, Grav1 Para1, 20 years old)

Even though they had been disappointed with services, they had also realised that some of the factors contributing to problems of care were employer-related such as insufficient staff.

‘They are short staffed, now we are being cared for by one sister and one nurse they can’t cope.’ (Participant 2, Grav2 Para1, 29 years old)

Staff shortages affect the quality of patient care and reduce staff morale as there is always not enough staff to engage with women (Bae et al. 2014:318; Bohren et al. 2015:17). Furthermore, nurses have a right to deliver safe patient care and therefore should not be jeopardised by short staffing, consumables or equipment (Eygelaar & Stellenberg 2012:10).

## Ethical considerations

Ethical clearance was granted by the NMMU (FRTI: No.H14-HEA-NUR-207). Further approvals were from the Department of Health (dated 19th January 2015) and managers at the respective hospitals and postnatal units utilised. Permission was also obtained from the participants. Ethical principles that were considered in the study were autonomy, beneficence and justice.

## Implications of the study

The findings of the study can contribute to midwifery training by highlighting the problems that mothers are faced with following a caesarean section delivery as well as the impact of these experiences in caring for their neonates. The findings will also raise awareness of both operational midwifery managers and midwives caring for these mothers following a caesarean section delivery of the physical and psychological stress that mothers are experiencing so that they can support and assist them more.

## Limitations of the study

The study focused on mother’s experiences in a selected institution; therefore, the study was not representative of the entire caesarean section deliveries in the Eastern Cape Province.

## Recommendations

The study recommends educating mothers about strategies for immediate self-care and neonatal care following a caesarean section delivery. The study also recommends that managers must address the administrative issues that contribute to the mothers’ experiences following a caesarean section delivery. Furthermore, the study recommends that another study be conducted in another municipal area to increase generalisation of the findings.

## Conclusion

From the findings of the study, it can be concluded that mothers had diverse experiences of pain; as a result they interpreted differently in terms of severity. Also, these experiences caused difficulty in performing self-care activities and caring for their neonates which was frustrating for the mothers. It was clear that mothers needed assistance and support regarding these self-care activities and breastfeeding. They also needed to be informed about hospital policies earlier in pregnancy so that they know what to expect.

## References

[CIT0001] Bae S., Kelly M., Brewer C.S. & Spencer A, 2014, ‘Analysis of nurse staffing and comprehensive nurse staffing characteristics in acute care nursing units’, *Journal of Nursing Care Quality* 29(4), 318–326. 10.1097/NCQ.0000000000000057 24509243

[CIT0002] Bamigboye A.A. & Hofmeyr G.J, 2012, ‘Caesarean section wound infiltration with local anaesthetic for postoperative pain relief- any benefit?’, *South African Medical Journal* 100(5), 313–319. 10.7196/SAMJ.3716 20460027

[CIT0003] Bohren M.A., Vogel J.P., Hunter E.C., Lutsiv O., Makh SK., Souza J.P. et al., 2014, ‘The maltreatment of women during childbirth in health facilities globally: A mixed-methods systematic review’, *PLOS Medicine Journal* 12(6), 1–32. 10.1371/journal.pmed.1001847 PMC4488322 26126110

[CIT0004] Botma Y., Greeff F.M., Mulaudzi F.M. & Wright S.C.D, 2010, *Research in health sciences*, Heinemann, Cape Town.

[CIT0005] Boutsikou T. & Malamitsi-Puchner A, 2011, ‘Caesarean section: Impact on mother and child’, *Acta Paediatrica* 100, 1518–1522. 10.1111/j.1651-2227.2011.02477.x 21950660

[CIT0006] Brink H., Van der Walt C. & Van Rensburg G, 2012, *Fundamentals of research methodology for healthcare professionals*, 3rd edn, Juta, Cape Town.

[CIT0007] Burns N. & Grove S.K, 2011, *Understanding nursing research-building an evidence based practice*, 5th edn, Elsevier Saunders, Maryland Heights, MO.

[CIT0008] Changee F., Iragpour F., Subar M. & Akbari S, 2015, ‘Client satisfaction of maternity care’, *In Lorestan Province Iran* 20(3), 398–404. PMC4462067 26120342

[CIT0009] Chin E.G., Vincent C. & Wilkie D, 2014, ‘A comprehensive description of postpartum pain after caesarean section delivery’, *Journal of Obstetric, Gynecologic & Neonatal Nursing* 43(6), 729–741. 10.1111/1552-6909.12483 25123692

[CIT0010] Creswell J.W, 2013, *Research design qualitative, quantitative and mixed methods: Approaches*, 3rd edn, Sage, Thousand Oaks, CA.

[CIT0011] Crowe L., Chang A., Fraser J.A., Gaskill D., Nash R. & Wallace K, 2008, ‘Systematic review of the effectiveness of nursing interventions in reducing post-operative pain’, *International Journal of Evidence Based Healthcare* 6(4), 396–430. 21631835 10.1111/j.1744-1609.2008.00113.x

[CIT0012] Declerq E., Cunningham D.K., Thomson C. & Salaka C, 2008, ‘Mothers reports of post-partum pain with vaginal and caesarean deliveries: Results of a national survey’, *Birth* 35(1), 16–24. 10.1111/j.1523-536X.2007.00207.x 18307483

[CIT0013] De Vos A.S., Strydom H., Fouché C.B. & Delport C.S.L, 2011, *Research at grass roots: For the social sciences and human service professionals*, 4th edn, Van Schaik, Pretoria.

[CIT0014] Dippenaar J. & Da Serra D, 2013, *Seller’s midwifery*, 2nd edn, Juta and Co Ltd., Lansdowne.

[CIT0015] Eygelaar J.E. & Stellenberg E.L, 2012, ‘Barriers to quality patient care in rural district hospitals’, *Academic Journal Curationis* 35(1), 1–7. 10.4102/curationis.v35i1.36 23327761

[CIT0016] Flick U, 2009, *An introduction to qualitative research*, 4th edn, Sage Publications, London.

[CIT0017] Freedman L.P., Ramsey K., Abuya T., Bellows B., Ndwiga C., Warren C.E. et al., 2014, ‘Defining disrespect and abuse of women in childbirth: A research, policy and rights agenda’, *Bulletin of World Health Organization* 92, 215–217. 10.2471/BLT.14.137869 PMC4264393 25552776

[CIT0018] Ismail S., Shahzad K. & Shafiq F, 2012, ‘Observational study to assess the effectiveness of postoperative pain management of patients undergoing elective cesarean section’, *Journal of Anaesthesiology, Clinical Pharmacology* 28(1), 36 10.4103/0970-9185.92432 PMC3275968 22345943

[CIT0019] Karim S.M.T., Goni M.R. & Murad M.H, 2013, ‘Medical negligence laws and patient safety in Bangladesh: An analysis’, *Journal of Alternative Perspective in the Social Science* 3(2), 424–442.

[CIT0020] Karlstrom A., Engstrom-Olofsson R., Nystedt A., Sjoling M. & Hildingsson I, 2010, ‘Women’s postoperative experiences before and after the introduction of spinal opioids in anaesthesia for caesarean section’, *Journal of Clinical Nursing* 19(9/10), 1326–1334. 10.1111/j.1365-2702.2010.03213.x 20500342

[CIT0021] Keedle H., Schmied V., Burns E. & Dahlen H.G, 2015, ‘Women’s reasons for, and experiences of choosing a homebirth following a caesarean section’, *BMC Pregnancy and Childbirth* 15(206), 1–12. 10.1186/s12884-015-0639-4 26337330 PMC4560080

[CIT0022] Kruger L.-M. & Schoombee C, 2010, ‘The other side of caring: Abuse in a South African maternity ward’, *Journal of Reproductive and Infant* 28(1), 84–107.

[CIT0023] Kuguoglu S., Yildiz H., Tanir M.K. & Demibarg B.C, 2012, ‘Breastfeeding after cesarean section’, in Salim R. (ed.), *Cesarean section*, pp. 121–160, InTech, Rijeka.

[CIT0024] Kumar R, 2014, *Research methodology: A step by step guide for beginners*, 4th edn, Sage, Los Angeles, CA.

[CIT0025] Lumadi T.G. & Buch E, 2011, ‘Patients’ satisfaction with midwifery services at a regional hospital in the Limpopo province of South Africa’, *Africa Journal of Nursing and Midwifery* 13(2), 14–28.

[CIT0026] McGrath P. & Phillips E, 2009, ‘The breast or bottle: Women’s infant feeding choices in a subsequent birth after a previous caesarean section’, *Journal of Advanced Nursing* 27(1), 37–47.

[CIT0027] Merriam S.B, 2009, *Qualitative research. A guide to design and implementation*. Jossey-Bass, San Francisco, CA.

[CIT0028] Monticelli F, 2013, ‘Caesarean section deliveries in public sector hospitals in South Africa, 2001–2009’, unpublished master’s research report, University of Witwatersrand, Johannesburg, Gauteng, South Africa.

[CIT0029] Mosadegharad A.M, 2014, ‘Factors influencing/affecting health service quality’, *International Journal of Health Policy Management* 3(2), 77–89. https://doi.org/10.15171/ijhpm.2014.65 25114946 10.15171/ijhpm.2014.65 PMC4122083

[CIT0030] Moyer C.A., Adongo P.B., Aborigo R.A., Hodgson A. & Engmann C.M, 2014, ‘They treat you like you are not a human being: Maltreatment during labour and delivery in rural northern Ghana’, *Midwifery* 30(2), 262–268. 10.1016/j.midw.2013.05.006 23790959

[CIT0031] Pattinson R.C. & Rhoda N, 2014, *Saving babies 2012–2013: Ninth report on perinatal care in South Africa*, Tshepesa Press, Pretoria, pp. 2–34.

[CIT0032] Polit D.F. & Beck C.T, 2012, *Nursing research. Generating and assessing evidence for nursing practice*, 9th edn, Wolters Kluwer, New York.

[CIT0033] Pound C.M. & Unger S.L, 2012, ‘The baby-friendly initiative: Protecting, promoting and supporting breastfeeding’, *Canadian Paediatric Society* 17(6), 317–321. PMC3380749 23730170

[CIT0034] Rauch E, 2011, ‘Intrathecal hydromorphone for cesarean delivery: In search of improved postoperative pain management: A case report’, *American Association of Nurse Anesthetists* 79(5), 427–432. 23256273

[CIT0035] Redshaw M, 2010, ‘Institutional processes and individual responses: Women’s experiences of care in relation to caesarean birth’, *Birth Issues in Perinatal Care* 37(2), 150–159. 10.1111/j.1523-536X.2010.00395.x 20557538

[CIT0036] Schmidt N.A. & Brown J.M, 2012, *Evidence-based practice for nurses: Appraisal and application of research*, 2nd edn, Jones & Bartlett, London.

[CIT0037] Sellers P.M, 2012, *Seller’s midwifery*, Juta, Cape Town.

[CIT0038] Sharma R., Artkin H., Mackillop L. & Paterson-Brown S, 2012, ‘Assessment of mobility of mothers postpartum to identify those at greatest risk of venous thromboembolism’, *Journal of Obstetric Gynaecology* 32(5), 461–463. 10.3109/01443615.2012.676694 22663319

[CIT0039] Soanes C. & Stevenson A. (eds.), 2006, *Concise Oxford English dictionary*, 11th edn, Oxford University Press, Oxford.

[CIT0040] Soanes C. & Stevenson A. (eds.), 2008, *Concise Oxford English dictionary*, 11th edn, Oxford University Press, Oxford.

[CIT0041] Van Reenen S.L. & Van Rensburg E, 2013, ‘The influence of an unplanned caesarean section on initial mother–infant bonding: Mothers subjective experiences’, *Journal of Psychology in Africa* 23(2), 269–274.

[CIT0042] Vercauteren M, 2010, ‘Analgesia after caesarean section delivery: Are neuraxical techniques outdated’, *Journal of Anaesthesia* 16(2), 129–133.

[CIT0043] Weiss M., Fawcett J. & Aber C, 2009, ‘Adaptation, postpartum concerns, and learning needs in the first two weeks after caesarean birth’, *Journal of Clinical Nursing* 18(21), 2938–2948. 10.1111/j.1365-2702.2009.02942.x 19821872

[CIT0044] World Health Organization & UNICEF, 2009, *Baby-friendly hospital initiative: revised, updated and expanded for integrated care. Section 1, Background and implementation*, viewed n.d., from http://apps.who.int/iris/bitstream/10665/43593/1/9789241594967_eng.pdf 23926623

[CIT0045] Zwedberg S., Blomquist J. & Sigterstad E, 2014, ‘Midwives’ experiences with mother-infant- skin-to-skin contact after a caesarean section: “Fighting an uphill battle”’, *Midwifery Journal* 31(1), 215–220. 10.1016/j.midw.2014.08.014 25241170

